# Characterization of unconventional kinetochore kinases KKT10 and KKT19 in *Trypanosoma brucei*

**DOI:** 10.1242/jcs.240978

**Published:** 2020-04-29

**Authors:** Midori Ishii, Bungo Akiyoshi

**Affiliations:** Department of Biochemistry, University of Oxford, Oxford OX1 3QU, UK

**Keywords:** Kinetoplastid kinetochore, *Trypanosoma brucei*, Kinase, KKT, Cell cycle

## Abstract

The kinetochore is a macromolecular protein complex that drives chromosome segregation in eukaryotes. Unlike most eukaryotes that have canonical kinetochore proteins, evolutionarily divergent kinetoplastids, such as *Trypanosoma brucei*, have unconventional kinetochore proteins. *T. brucei* also lacks a canonical spindle checkpoint system, and it therefore remains unknown how mitotic progression is regulated in this organism. Here, we characterized, in the procyclic form of *T. brucei*, two paralogous kinetochore proteins with a CLK-like kinase domain, KKT10 and KKT19, which localize at kinetochores in metaphase but disappear at the onset of anaphase. We found that these proteins are functionally redundant. Double knockdown of KKT10 and KKT19 led to a significant delay in the metaphase to anaphase transition. We also found that phosphorylation of two kinetochore proteins, KKT4 and KKT7, depended on KKT10 and KKT19 *in vivo*. Finally, we showed that the N-terminal part of KKT7 directly interacts with KKT10 and that kinetochore localization of KKT10 depends not only on KKT7 but also on the KKT8 complex. Our results reveal that kinetochore localization of KKT10 and KKT19 is tightly controlled to regulate the metaphase to anaphase transition in *T. brucei*.

This article has an associated First Person interview with the first author of the paper.

## Abstract (precis)

**Summary:** Trypanosomes lack a canonical spindle checkpoint system, but kinetochore kinases KKT10/19 phosphorylate KKT4 and KKT7 and are important for promoting the metaphase to anaphase transition in *T. brucei.*

## INTRODUCTION

Proper segregation of chromosomes into two daughter cells during cell division is essential for the survival of all eukaryotes. Chromosomes are replicated during S phase and linked together by cohesin complexes (Nasmyth and Haering, 2009). The kinetochore is a macromolecular protein complex, which attaches to the centromeric region of each chromosome and interacts with spindle microtubules (Cheeseman, 2014; Santaguida and Musacchio, 2009). Kinetochore–microtubule attachments are monitored by a feedback mechanism called the spindle checkpoint, which delays the metaphase to anaphase transition until all chromosomes are attached to spindle microtubules emanating from opposite poles (Hoyt et al., 1991; Li and Murray, 1991). Spindle checkpoint components, such as Mad1, Mad2, Mad3/BubR1, Bub1, Bub3 and Mps1 are recruited to unattached kinetochores, creating a signal that inhibits Cdc20, an activator of the ubiquitin ligase called the anaphase promoting complex/cyclosome (APC/C) (Musacchio and Salmon, 2007). Once all chromosomes are properly bi-oriented, the spindle checkpoint is satisfied and the APC/C gets activated, which leads to the degradation of securin and cyclin B (Yamano, 2019). Degradation of securin leads to the cleavage of cohesin complexes and separation of sister chromatids (Nasmyth et al., 2000; Yanagida, 2000), while that of cyclin B promotes mitotic exit (Hershko, 1999).

Kinetochores in many eukaryotes consist of more than 40 different proteins, some of which are conserved even in diverse eukaryotes (Meraldi et al., 2006; van Hooff et al., 2017). However, none of canonical kinetochore proteins is found in a group of evolutionarily divergent eukaryotes called kinetoplastids (Berriman et al., 2005; Lowell, 2004). In *T. brucei*, which is a kinetoplastid parasite that causes human African trypanosomiasis (sleeping sickness) in sub-Saharan Africa, a number of unique kinetochore proteins have been identified, including KKT1–KKT20, KKT22–KKT25, and KKIP1–KKIP12 (Akiyoshi and Gull, 2014; Brusini et al., 2019 preprint; D'Archivio and Wickstead, 2017; Nerusheva and Akiyoshi, 2016; Nerusheva et al., 2019). Furthermore, homologs of spindle checkpoint components are apparently absent in *T. brucei*, except for a Mad2-like protein. However, this protein localizes only at basal bodies, and Cdc20 does not have a well-conserved Mad2-binding motif (Akiyoshi and Gull, 2013). Consistent with these findings, depolymerization of spindle microtubules does not delay the metaphase-to-anaphase transition (Ploubidou et al., 1999), suggesting that *T. brucei* indeed lacks a canonical spindle checkpoint system. In contrast, *T. brucei* has functional homologs of other mitotic machineries, such as the CDK/cyclin system (Hammarton et al., 2003; Tu and Wang, 2004) and the anaphase promoting complex/cyclosome (APC/C) (Kumar and Wang, 2005). It remains unclear whether there is any regulatory mechanism for mitotic progression in *T. brucei*.

Protein kinases are known to play regulatory roles at various cellular locations, including kinetochores. Among known kinetoplastid kinetochore proteins, four proteins have a kinase domain, namely KKT2, KKT3, KKT10 and KKT19 (Akiyoshi and Gull, 2014). Because these proteins are not present in humans, they are attractive drug targets against kinetoplastid parasites. Previous studies have identified small molecules that inhibit KKT10 and KKT19 (also known as TbCLK1 and TbCLK2; hereafter KKT10/19) (Nishino et al., 2013; Saldivia et al., 2019 preprint; Torrie et al., 2019). KKT10/19 are paralogous proteins, apparently made by a recent gene duplication event (Akiyoshi and Gull, 2014). Their kinase domain is 100% identical and is classified as a member of the cdc2-like (CLK) kinase subfamily (Parsons et al., 2005). Although inhibition of KKT10/19 by RNAi-mediated knockdown or chemical compounds severely affects cell growth (Akiyoshi and Gull, 2014; Alsford et al., 2011; Jones et al., 2014; Nishino et al., 2013; Saldivia et al., 2019 preprint), little is known about their molecular functions.

Here, we have characterized KKT10/19 in *T. brucei*. We show that they are functionally redundant in procyclic form cells. Their double knockdown causes a delay in the metaphase-to-anaphase transition without affecting the localization of other kinetochore proteins. We also show that KKT4 and KKT7 are phosphorylated in a KKT10/19-dependent manner and identify KKT4 as a key substrate. Furthermore, we identify KKT7 as a direct interaction partner of KKT10. However, kinetochore localization of KKT10 depends not only on KKT7 but also on the KKT8 complex. Taken together, our data reveal that KKT10/19 play essential regulatory roles in trypanosomes.

## RESULTS

### KKT10 and KKT19 are functionally redundant

**Fig. 1.**
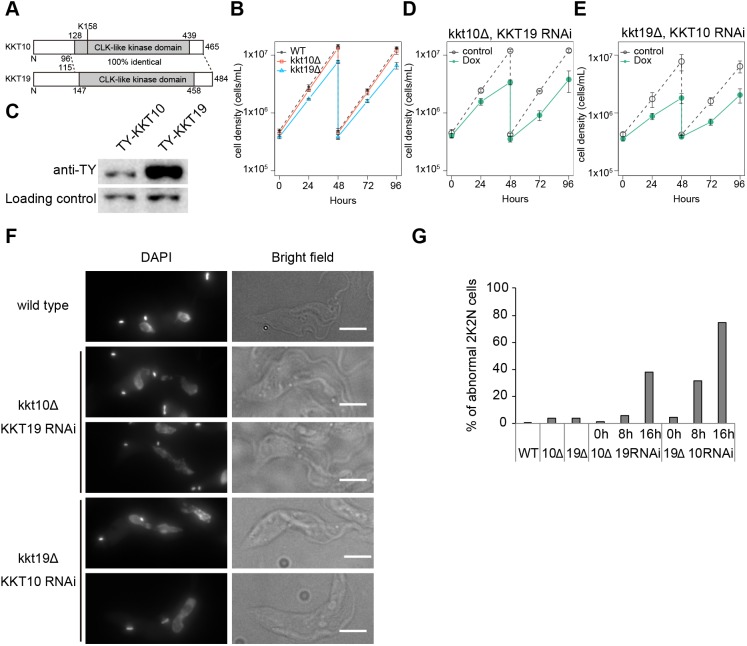
**KKT10 and KKT19 are redundant.** (A) Schematic representation of KKT10 and KKT19 proteins. (B) KKT10 (red) and KKT19 (blue) knockout cells are viable. Gray dashed line indicates a WT control. Results are mean±s.d. from three independent experiments. Similar results were obtained from at least two independent clones. (C) Protein levels of TY-YFP-tagged KKT10 and KKT19 were monitored by immunoblotting. Representative of three independent experiments is shown. PFR2 was used as a loading control. Uncropped images are shown in Fig. S1. (D,E) RNAi-mediated knockdown of (D) KKT19 in kkt10 deletion cells and (E) KKT10 in kkt19 deletion cells affects cell growth. Control is an uninduced cell culture. Results are mean±s.d. from three independent experiments. (F,G) KKT10/19 double depletion causes chromosome missegregation. (F) Examples of anaphase cells fixed at 16 h post induction of RNAi and stained with DAPI. Note that in WT cells, daughter nuclei appear as a smooth shape without significant lagging DNA in between them. In contrast, lagging chromosomes and/or abnormal nuclear signals were often observed upon depletion of KKT10/19. Maximum intensity projections are shown. Scale bars: 5 µm. (G) Quantification of the percentage of 2K2N cells with abnormal chromosome segregation (*n*≥74).

Previous studies have shown that simultaneous knockdown of both KKT10/19 causes growth defects in procyclic (Akiyoshi and Gull, 2014) and bloodstream form cells (Jones et al., 2014; Nishino et al., 2013; Saldivia et al., 2019 preprint). Although KKT10/19 have an identical protein kinase domain (Fig. 1A), it remained unclear whether they have distinct functions. Previous studies have shown that KKT10 is essential for the proliferation of bloodstream form cells, whereas KKT19 was not (Jones et al., 2014; Nishino et al., 2013; Saldivia et al., 2019 preprint). To investigate their functions in procyclic cells, we performed RNAi using a construct that specifically targeted KKT10. We first confirmed reduction of the YFP–KKT10 signal upon induction of RNAi (Fig. S1A). However, to our surprise, these KKT10-depleted cells grew normally (Fig. S1B). We obtained a similar result for KKT19 depletion (data not shown), suggesting that KKT10 and KKT19 may be functionally redundant in procyclic cells. To test this possibility, we made strains that lacked KKT10 or KKT19. Both alleles of KKT10 or KKT19 coding regions were replaced with drug resistant gene cassettes by a PCR-based method (Merritt and Stuart, 2013) to make kkt10Δ/kkt10Δ (kkt10Δ) or kkt19Δ/kkt19Δ (kkt19Δ) cells. Consistent with our RNAi results, both kkt10Δ and kkt19Δ cells were viable, confirming that KKT10 and KKT19 are functionally redundant in procyclic cells. However, kkt19Δ cells grew more slowly than wild-type or kkt10Δ cells (Fig. 1B). We found that KKT19 proteins are more abundant than KKT10 in wild-type cells (Fig. 1C; Fig. S1C). The mild growth defect of kkt19Δ cells may therefore imply that the amount of KKT10 protein is insufficient to ensure normal cell growth.

To deplete both KKT10 and KKT19 proteins, we performed KKT19-specific RNAi in kkt10Δ cells (Fig. 1D) or KKT10-specific RNAi in kkt19Δ cells (Fig. 1E), and found severe growth defects in both cases. To investigate the phenotype of KKT10 and KKT19 depletion, we examined chromosome segregation in anaphase. As we previously showed in KKT10/19 double-knockdown cells (Akiyoshi and Gull, 2014), kkt10Δ KKT19 RNAi and kkt19Δ KKT10 RNAi cells had abnormal chromosome segregation in anaphase cells (Fig. 1F,G). In contrast, kkt10Δ or kkt19Δ cells had only small numbers of mis-segregated chromosomes (Fig. 1G). These results confirm that KKT10/19 are functionally redundant in procyclic cells.

### Metaphase to anaphase transition is delayed without KKT10/19

**Fig. 2.**
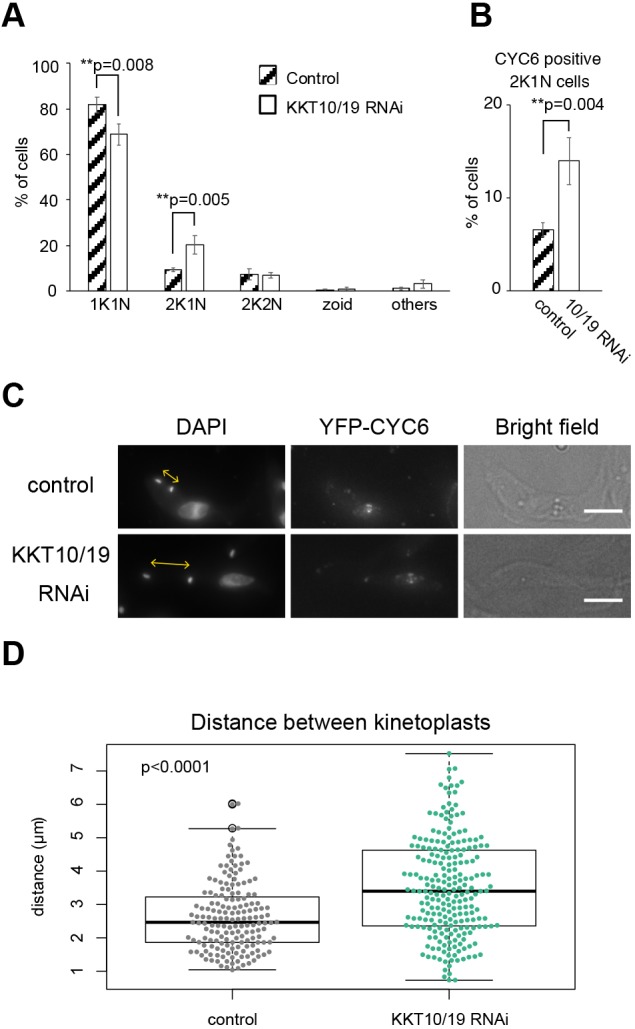
**KKT10/19 depletion delays the metaphase to anaphase transition.** (A,B) Quantification of (A) cells with indicated DNA contents, or (B) 2K1N cells that have nuclear CYC6 signals. Cells were fixed at 24 h post induction of KKT10/19 RNAi. Control is an uninduced cell culture. *P*-values were calculated by Student's *t*-test, one-tailed distribution. Results are mean±s.d. from three independent experiments (*n*≥246). (C) KKT10/19-depleted cells have a longer distance between kinetoplasts. Cells were fixed at 24 h post induction of RNAi and stained with DAPI. Control is an uninduced cell culture. Maximum intensity projections are shown. Scale bars: 5 µm. (D) Quantification of distance between two kinetoplasts in nuclear CYC6-positive 2K1N cells upon KKT10/19 RNAi. The box represents the 25–75th percentiles, and the median is indicated. The whiskers show the 1.5× interquartile range. Data were collected from cells at 24 h post induction. Control is an uninduced cell culture. *P*<0.0001 was calculated by Welch two sample *t*-test (*n*≥167).

KKT10/19 localize at kinetochores from S phase until anaphase onset. This localization pattern is reminiscent of that of spindle checkpoint proteins in other eukaryotes despite the fact that *T. brucei* does not appear to have a functional spindle checkpoint (Hayashi and Akiyoshi, 2018; Ploubidou et al., 1999). Although it has been shown that inhibition of KKT10/19 results in cell cycle defects in bloodstream form cells (Jones et al., 2014; Saldivia et al., 2019 preprint), it remained unclear whether KKT10/19 have a direct role in cell cycle regulation. To address this question, we examined the cell cycle status of KKT10/19 knockdown cells (Fig. S2A) (Akiyoshi and Gull, 2014). *T. brucei* has a characteristic DNA structure called the kinetoplast, which contains mitochondrial DNA. Kinetoplasts segregate prior to the nuclear division, thus the number of kinetoplasts (K) and nuclei (N) in a cell indicates the cell cycle stage: 1K1N (one kinetoplast and one nucleus) for G1 to S phase, 2K1N (two kinetoplasts and one nucleus) for G2 to metaphase, and 2K2N (two kinetoplasts and two nuclei) for anaphase to telophase (Robinson et al., 1995). We found that the ratio of 1K1N cells decreased, while that of 2K1N cells increased in KKT10/19 knockdown cells at 24 h post induction (Fig. 2A). We also analyzed the cell cycle profile in kkt10Δ KKT19 RNAi and kkt19Δ KKT10 RNAi cells, and obtained similar results (Fig. S2B,C). These results suggest that there is a delay in nuclear division upon depletion of KKT10/19. To directly test this possibility, we monitored YFP-tagged cyclin B (CYC6), which appears in the nucleus in G2 and disappears at the onset of anaphase (Hayashi and Akiyoshi, 2018). We found that the number of nuclear CYC6-positive 2K1N cells increased in KKT10/19 knockdown cells (Fig. 2B), confirming the delay in the metaphase-to-anaphase transition.

In *T. brucei*, the distance between two kinetoplasts is another cell cycle marker, which gets longer as cell cycle progresses and becomes maximum before cytokinesis (Robinson et al., 1995). Although nuclear and cytoplasmic events are coordinated under proliferating conditions, inhibition of nuclear division does not prevent the progression of cytoplasmic events (Ploubidou et al., 1999). We therefore measured the distance between the two kinetoplasts in 2K1N (G2/M) cells that have nuclear CYC6 signals to examine their cytoplasmic cell cycle status (Fig. 2C,D). The average distance between the two kinetoplasts was 2.7 µm in control cells compared to 3.5 µm in KKT10/19 knockdown cells (Fig. 2D). This result further supports our finding that the metaphase to anaphase transition in the nucleus is delayed in KKT10/19 knockdown cells.

### KKT10/19 are dispensable for the localization of other kinetochore proteins

**Fig. 3.**
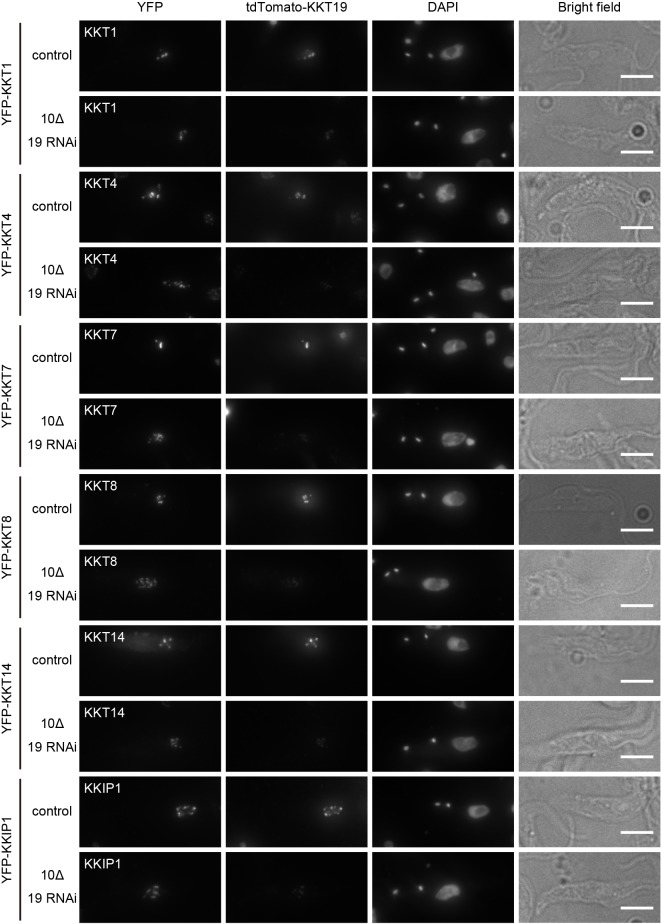
**KKT10/19 are dispensable for the localization of other kinetochore proteins.** YFP-tagged KKT1, 4, 7, 8, 14 or KKIP1 were imaged after KKT10/19 depletion. Examples of 2K1N cells fixed at 24 h post induction of RNAi and stained with DAPI are shown. Control is an uninduced cell culture. Maximum intensity projections are shown. Scale bars: 5 µm. Similar results were obtained in all 2K1N cells (*n*≥35).

It was recently shown that treatment of bloodstream form cells with AB1, a covalent kinase inhibitor against KKT10, affected the localization of some kinetochore proteins (Saldivia et al., 2019 preprint). Given its potential off-target effects, however, it remains unclear whether the observed defects were actually due to inhibition of KKT10/19. To test whether KKT10/19 regulate the localization of kinetochore proteins in procyclic cells, YFP-tagged KKT1, KKT4, KKT7, KKT8, KKT14 and KKIP1 were imaged in kkt10Δ KKT19 RNAi cells (Fig. 3). These proteins localize at kinetochores at different cell cycle stages in wild-type cells. KKT4, a microtubule-binding component, localizes at kinetochores constitutively (Akiyoshi and Gull, 2014; Llauró et al., 2018). KKT1, KKT7 and KKIP1 localize at kinetochores from S phase to anaphase, while KKT8 localizes from S to metaphase (Akiyoshi and Gull, 2014; D'Archivio and Wickstead, 2017). KKT14 localizes from G2 to anaphase (Akiyoshi and Gull, 2014). We found that although severe chromosome segregation defects were observed, kinetochore localization of these proteins was not perturbed by KKT10/19 knockdown (Fig. 3). Therefore, KKT10/19 are not essential for the localization of these kinetochore proteins in procyclic cells.

### Kinase activity of KKT10 is essential for cell proliferation

**Fig. 4.**
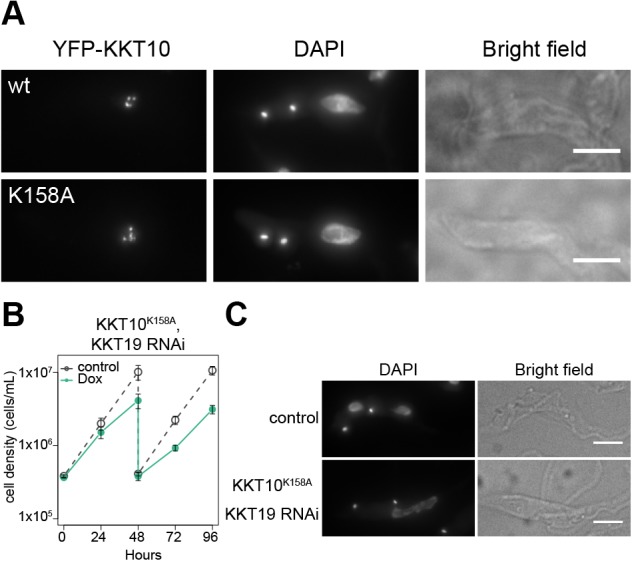
**Kinase activity of KKT10 is essential for cell proliferation.** (A) Examples of cells expressing YFP-KKT10 (WT or K158A mutant) stained with DAPI. Maximum intensity projections are shown. (B) Expression of YFP–KKT10^K158A^ fails to rescue the KKT19 RNAi phenotype. Control is an uninduced cell culture. Results are mean±s.d. from three independent experiments. Similar results were obtained from three clones. (C) Cells expressing YFP–KKT10^K158A^ in the kkt10Δ KKT19 RNAi strain were fixed at 24 h post induction of RNAi and stained with DAPI. Control is an uninduced cell culture. Maximum intensity projections are shown. Scale bars: 5 µm.

To investigate the importance of KKT10/19 kinase activities, we made a kinase-dead form of KKT10 by mutating lysine 158 (Fig. 1A), a residue conserved in active protein kinases in eukaryotes, in the N-terminal YFP-tagging construct for KKT10. We first confirmed that wild-type YFP–KKT10 (YFP–KKT10^WT^) was able to rescue the KKT10/19 double knockdown phenotype (Fig. S3A). We then made a strain that had YFP–KKT10^K158A^ as the sole source of KKT10 in cells. YFP–KKT10^K158A^ localized normally at kinetochores from S phase to metaphase (Fig. 4A). However, we observed a severe growth defect upon depletion of KKT19 (Fig. 4B), which was almost comparable to that seen upon the double knockdown of KKT10/19 (Fig. 1D,E). As expected, abnormal chromosome segregation was observed in KKT10 kinase-dead cells (Fig. 4C). These results show that the kinase activity is important for the function of KKT10 but is not necessary for its own kinetochore localization.

### Phosphorylation of KKT4 and KKT7 depends on KKT10/19

**Fig. 5.**
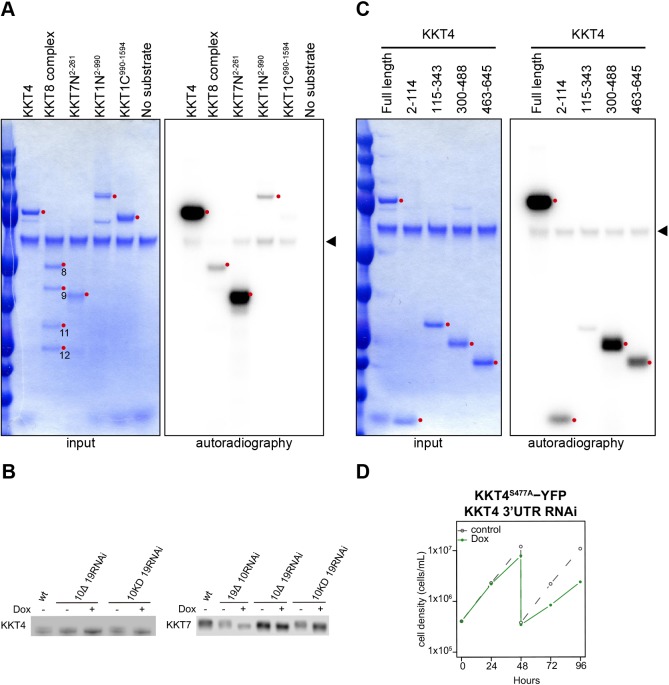
**KKT10 phosphorylates KKT4 and KKT7 *in vitro* and *in vivo*.** (A) KKT10 *in vitro* kinase assay using the indicated recombinant proteins as substrates. The left panel (input) shows the Coomassie Brilliant Blue staining. Phosphorylation was detected by autoradiography. The arrowhead indicates KKT10. (B) Phosphorylation of KKT4 and KKT7 depends on KKT10/19. 3Flag-tagged KKT4 and KKT7 were detected upon induction of RNAi for 24 h. 10 KD is KKT10^K158A^. Uncropped images are shown in Fig. S3. Images representative of at least three independent experiments are shown. (C) KKT10 *in vitro* kinase assay on KKT4 fragments. The left panel (input) shows the Coomassie Brilliant Blue staining. Phosphorylation was detected by autoradiography. Arrowhead indicates KKT10. (D) KKT4^S477A^–YFP fails to rescue the KKT4 3′UTR RNAi phenotype. Control is an uninduced cell culture. Similar results were obtained from two clones.

We next aimed to identify the target of KKT10/19 kinases. A number of phosphorylation sites on kinetochore proteins have been detected by mass spectrometry of *T. brucei* cell extracts and immunoprecipitates of kinetochore proteins (Akiyoshi and Gull, 2014; Benz and Urbaniak, 2019; Nerusheva and Akiyoshi, 2016; Nerusheva et al., 2019; Nett et al., 2009; Urbaniak et al., 2013) (Tables S1–S3). To identify KKT10/19 substrates, we performed an *in vitro* kinase assay using several recombinant kinetochore proteins and found that KKT4, KKT8, KKT7^2-261^ and KKT1^2-990^ were phosphorylated by KKT10 (Fig. 5A). Among these four proteins, KKT4 and KKT7 were the most strongly phosphorylated, so we next tested whether their phosphorylation depends on KKT10/19 *in vivo* by performing immunoblots against these proteins. We detected electrophoretic mobility shifts of KKT4 and KKT7 in wild-type cells, which disappeared in KKT10/19-depleted cells and KKT10 kinase-dead cells (Fig. 5B; Fig. S3B,C). These results show that KKT4 and KKT7 are phosphorylated in a KKT10/19-dependent manner *in vivo*.

To further investigate the phosphorylation of KKT4, we dissected KKT4 into four fragments (residues 2–114, 115–343, 300–488 and 463–645). We previously showed that KKT4^115–343^ binds microtubules *in vitro* (Llauró et al., 2018), but this fragment was not robustly phosphorylated by KKT10 (Fig. 5C). Instead, the KKT4^300–488^ fragment showed the strongest phosphorylation by KKT10 in this assay (Fig. 5C). Our sequence analysis identified three serine residues (S334, S463, and S477) in KKT4^300–488^ that match the consensus phosphorylation motif of KKT10/19 (RxxS) (Torrie et al., 2019). Among these sites, S334 and S477 have been shown to be phosphorylated *in vivo* (Urbaniak et al., 2013). To test the relevance of their phosphorylation *in vivo*, we made a phospho-deficient mutant (KKT4^S334A S463A S477A^) tagged with a C-terminal YFP, and found that it failed to rescue the growth defect caused by the KKT4 3′UTR RNAi, whereas KKT4^WT^–YFP rescued the defect (Fig. S4A). We next made individual phospho-deficient mutants (KKT4^S334A^, KKT4^S463A^ and KKT4^S477A^) with a C-terminal YFP tag and tested their function. While KKT4^S334A^ and KKT4^S463A^ rescued the growth defect of the KKT4 3′UTR RNAi (Fig. S4A), KKT4^S477A^ failed to do so (Fig. 5D), suggesting that phosphorylation of S477 plays an important role for the function of KKT4. By contrast, we did not observe any growth defect for the KKT7^10A^ protein that has all the serine residues that match the KKT10/19 consensus phosphorylation motif mutated to alanine residues (S23A, S34A, S36A, S65A, S148A, S150A, S257A, S263A, S317A, S490A) (Fig. S5A), suggesting that the KKT10/19-dependent phosphorylation of KKT7 is dispensable for the proliferation of procyclic cells. Importantly, all the phospho-deficient mutants of KKT4 and KKT7 we made localized normally at kinetochores (Figs S4B and S5B), consistent with our finding that KKT10/19-depletion did not affect their kinetochore localization (Fig. 3).

### N-terminal part of KKT7 recruits KKT10/19 onto kinetochores

**Fig. 6.**
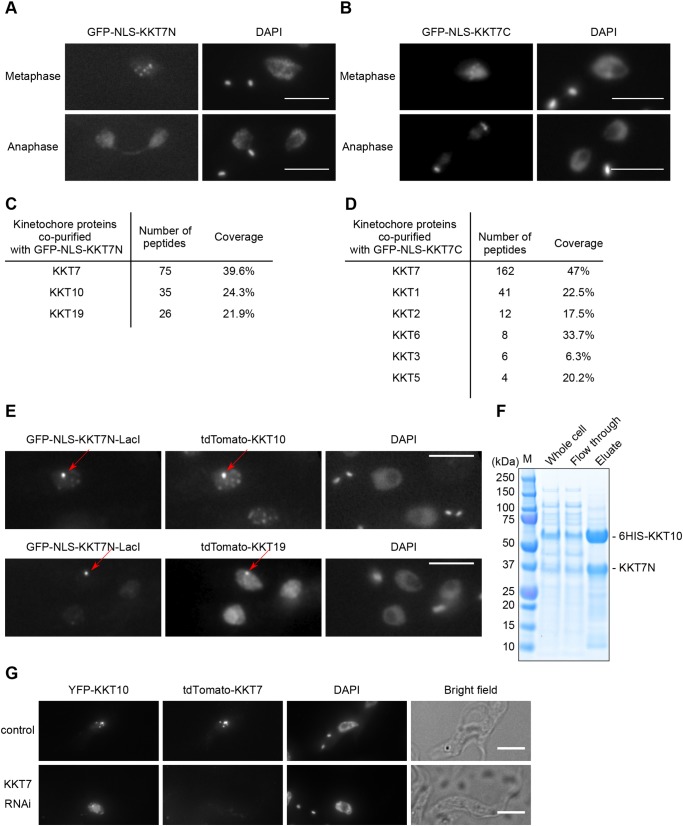
**KKT7 binds and recruits KKT10/19 onto kinetochores.** (A) Ectopically expressed GFP-NLS-KKT7N^2–261^ localizes at kinetochores in metaphase, but not in anaphase. (B) Ectopically expressed GFP-NLS-KKT7C^262–644^ localizes at kinetochores in metaphase and anaphase. (C) KKT7N^2–261^ co-purifies with KKT10 and KKT19, but not with other kinetochore proteins. See Table S4 for all proteins identified by mass spectrometry. (D) KKT7C^262–644^ co-purifies with several kinetochore proteins, but not with KKT10/19. See Table S5 for all proteins identified by mass spectrometry. (E) KKT7N^2–261^ is sufficient to recruit KKT10 and KKT19 to a non-centromeric locus *in vivo*. For A–E, inducible GFP fusion proteins were expressed with 10 ng/ml doxycycline for 24 h. (F) KKT7N^2–261^ directly interacts with KKT10. Recombinant KKT7N^2–261^ and 6HIS–KKT10 proteins were co-expressed in *E. coli*, followed by metal affinity chromatography. (G) Localization of YFP-tagged KKT10 is affected in KKT7-knockdown cells. Similar results were obtained in 88% of 2K1N cells (*n*=26). Cells were fixed at 24 h post induction of RNAi and stained with DAPI. Control is an uninduced cell culture. Maximum intensity projections are shown. Scale bars: 5 µm.

We next aimed to reveal how KKT10/19 are recruited to kinetochores and how their localization is regulated. Our previous data showed that KKT7 was one of the most abundant proteins that co-purified with YFP-tagged KKT10 or KKT19 (Akiyoshi and Gull, 2014). During the course of our studies on KKT7, we found that its N-terminal region (residues 2–261; KKT7N) or the C-terminal region (262–644; KKT7C) can localize at kinetochores when ectopically expressed in wild-type trypanosomes (Fig. 6A,B). Immunoprecipitation of these fragments revealed that KKT7N co-purified with KKT10 and KKT19 (Fig. 6C; Table S4), while KKT7C co-purified with a number of kinetochore proteins, but not with KKT10 or KKT19 (Fig. 6D; Table S5). These results indicate that KKT7 is recruited to kinetochores via its C-terminal part and that KKT10 is recruited by the N-terminal part of KKT7. Kinetochore localization of KKT7N in metaphase, but not in anaphase when KKT10/19 disappear from kinetochores, supports this idea. To test whether the KKT7 N-terminal region is sufficient to recruit KKT10, we used a LacO-LacI system (Landeira and Navarro, 2007) to tether KKT7N to an ectopic locus. We found that KKT10 and KKT19 are recruited to the KKT7N–LacI protein on the LacO locus (Fig. 6E). We then tested whether KKT10 directly interacts with KKT7N. We co-expressed 6HIS–KKT10 and KKT7N in *E. coli* and performed metal affinity chromatography, revealing that KKT7N co-purifies with 6HIS–KKT10 (Fig. 6F). Finally, we examined whether the localization of KKT10 depends on KKT7. In control 2K1N cells, YFP–KKT10 appears as multiple dots with little diffuse nuclear signal (Fig. 6G) (Akiyoshi and Gull, 2014). In KKT7-depleted cells, however, we found that the YFP–KKT10 signal was mostly diffuse in the nucleus, occasionally with one or two bright dots (Fig. 6G). Although these bright dots may reflect residual kinetochore localization of KKT10, these data strongly suggest that the kinetochore localization of KKT10 is affected upon depletion of KKT7. Taken together, these results establish that KKT10/19 are recruited to the kinetochore by interacting with the N-terminal region of KKT7.

### Localization of KKT10 is also dependent on KKT9 and KKT11

**Fig. 7.**
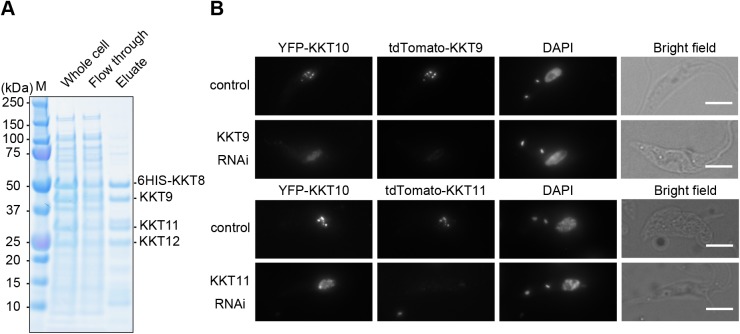
**KKT10 localization is also dependent on the KKT8 complex.** (A) KKT8, KKT9, KKT11 and KKT12 form a complex. Recombinant 6HIS-tagged KKT8, KKT9, KKT11 and KKT12 proteins were co-expressed in *E. coli*, followed by metal affinity chromatography. (B) Localization of YFP-tagged KKT10 is affected in KKT9 and KKT11 knockdown cells. Similar results were obtained in more than 75% of 2K1N cells (*n*≥16). Cells were fixed at 24 h post induction of RNAi and stained with DAPI. Control is an uninduced cell culture. Maximum intensity projections are shown. Scale bars: 5 µm.

Regulation of spatio-temporal localization of a protein is often linked with its functional regulation (Dou et al., 2019). Although both KKT7 and KKT10/19 start localizing at kinetochores from S phase, KKT10/19 disappear from kinetochores at the onset of anaphase, while KKT7 stays at kinetochores during anaphase (Akiyoshi and Gull, 2014). These observations imply that kinetochore localization of KKT10/19 is tightly regulated during the cell cycle. Interestingly, four other kinetochore proteins (KKT8, KKT9, KKT11 and KKT12) have a similar localization pattern to KKT10/19 (Akiyoshi and Gull, 2014). We used a bacterial co-expression system and found that KKT9, KKT11 and KKT12 co-purified with 6HIS–KKT8, suggesting that they form a complex (Fig. 7A). We call this four-protein complex the KKT8 complex. Given its similar localization pattern to KKT10/19, we next tested whether the KKT8 complex regulates the localization of KKT10 by performing RNAi against two of its components, KKT9 and KKT11, for which efficient depletions were achieved. We found that YFP–KKT10 failed to localize at kinetochores properly in both cases (Fig. 7B). Therefore, kinetochore localization of KKT10 relies not only on KKT7 but also the KKT8 complex.

To determine the localization hierarchy between KKT7 and the KKT8 complex, we examined the localization of KKT9 in KKT7 RNAi cells and vice versa. Kinetochore localization of YFP–KKT9 was affected in KKT7 RNAi cells, showing one or two bright blobs with diffuse nuclear signal (Fig. S6A). In contrast, YFP–KKT7 formed kinetochore-like dots after KKT9 RNAi (Fig. S6B). Taken together, these results show that KKT7 is required for the localization of both the KKT8 complex and KKT10, while the KKT8 complex is required for the localization of KKT10, but not KKT7. A corollary is that KKT7, despite its ability to directly bind KKT10, is unable to recruit KKT10 to kinetochores in the absence of the KKT8 complex *in vivo*, which is consistent with the fact that KKT10/19 disappear from kinetochores in anaphase when the KKT8 complex also disappears.

## DISCUSSION

Protein kinases play three major regulatory roles at kinetochores in eukaryotes: kinetochore assembly (e.g. CDKs and Aurora B), error correction (e.g. Aurora B and Mps1), and the spindle checkpoint (e.g. Mps1 and Bub1) (London and Biggins, 2014; Musacchio and Desai, 2017). While apparent homologs of Mps1 or Bub1 are absent, those for CDKs (Hammarton et al., 2003; Tu and Wang, 2004) and Aurora B (Li et al., 2008) are present in *T. brucei*. In addition, there are four protein kinases that specifically localize at kinetochores. KKT2 and KKT3, whose kinase domains are classified as unique among eukaryotic kinase subfamilies, are putative DNA-binding kinetochore proteins that localize at kinetochores throughout the cell cycle. In contrast, KKT10/19, which have a CLK-like kinase domain, localize at kinetochores from S phase until the onset of anaphase. In other eukaryotes, CLK kinases are implicated in RNA splicing (Corkery et al., 2015), not kinetochore functions, suggesting that KKT10/19 likely evolved from a CLK kinase to carry out distinct functions in kinetoplastids.

*T. brucei* has a complex life cycle to survive in both animal hosts and insect vectors, and cells change many biological features to adapt to different environments (Matthews, 2005). Our findings in procyclic cells show that KKT10 and KKT19 are functionally redundant and that KKT19 is more abundant than KKT10. In contrast, previous studies in bloodstream form cells have shown that KKT10 was essential for proliferation, while KKT19 is not, raising a possibility that KKT10 may be more abundant than KKT19 in this life stage. Alternatively, it is possible that KKT10 and KKT19 have non-overlapping functions in bloodstream form cells.

Saldivia et al. recently found that a covalent kinase inhibitor AB1 (Lelais et al., 2016) has potent activity against several trypanosomatids and identified KKT10 as a target in bloodstream form *T. brucei* (Saldivia et al., 2019 preprint). They also found that KKT10 phosphorylated KKT2 at serine 507 and/or serine 508, and that the KKT2^S507A S508A^ phospho-deficient mutant failed to rescue the growth defect caused by KKT2 RNAi in bloodstream form cells. They further showed the phosphorylation at these residues by KKT10 was important for recruiting some kinetochore proteins to kinetochores (Saldivia et al., 2019 preprint). However, we did not find significant localization defects of kinetochore proteins in KKT10/19-depleted procyclic cells. Moreover, the KKT2^S507A S508A^ mutant localized normally at kinetochores and supported cell growth upon induction of KKT2 RNAi (Fig. S7). We do not know the underlying molecular mechanisms for the observed differences between bloodstream and procyclic form cells. Further study is required to understand life stage-specific and common mechanisms of kinetochore assembly in *T. brucei*.

In other eukaryotes, the spindle checkpoint monitors attachment errors and delays mitotic progression by inhibiting Cdc20, an activator of APC/C. *T. brucei* lacks canonical spindle checkpoint components but has APC/C. Building on previous studies that noted cell cycle defects upon KKT10 knockdown in bloodstream form cells (Jones et al., 2014; Saldivia et al., 2019 preprint), we used cyclin B as a nuclear cell cycle marker and showed that KKT10/19 are important for the metaphase to anaphase transition in procyclic cells. Interestingly, one of their targets, KKT4, has microtubule-binding activities (Llauró et al., 2018) and co-purifies with some APC/C subunits (Akiyoshi and Gull, 2014). We speculate that phosphorylation of KKT4 may change depending on its microtubule attachment status, directly affecting the interaction between KKT4 and APC/C subunits. The molecular mechanism of this potentially new form of mitotic regulation will need to be investigated in the future for better understanding of cell cycle progression in *T. brucei*.

## MATERIALS AND METHODS

### Primers, plasmids, bacmids and synthetic DNA

Plasmids/bacmids, primers and synthetic DNA fragments used in this study are listed in Tables S6–S8, respectively. All constructs were sequence verified. To make head-to-head RNAi constructs (pBA188; KKT19-specific and pBA190; KKT10-specific), gene fragments (KKT19 26–338 bp and KKT10 60–286 bp) were amplified with primers BA557/BA558 (KKT19) and BA561/BA562 (KKT10) from genomic DNA, and cloned into p2T7-177 (Wickstead et al., 2002) using *Spe*I/*Hin*dIII. To make hairpin RNAi constructs (pBA865; KKT7, pBA1554; KKT7 5′UTR, pBA866; KKT10-specific, pBA1200; KKT11, and pBA1710; KKT2), synthetic DNA fragments [BAG28; KKT7 33–489 bp, BAG89; KKT7 5′UTR 374 bp, BAG29; KKT10 14–286 bp, BAG61; KKT11 99–598 bp, and BAG94; KKT2 5′UTR 342 bp (GeneArt)] were cloned into pBA310 (Nerusheva and Akiyoshi, 2016) using *Hin*dIII/*Bam*HI. To make pBA1356 (TY-YFP-KKT10), the N-terminal region of the KKT10 coding sequence (4–1000 bp) was amplified with primers BA294/BA1874 from genomic DNA, and cloned into pBA74 (Akiyoshi and Gull, 2014) using *Xba*I/*Not*I. To make pBA1357 (TY-YFP-KKT10^K158A^), site-directed mutagenesis was performed using primers BA1875/BA1876 and pBA1356 as a template. To make N-terminal TY-tdTomato-tagging constructs (pBA1373; KKT19, pBA1419; KKT7, pBA1420; KKT8, pBA1586; KKT9 and pBA1587; KKT11), endogenous gene targeting sequences from TY-YFP-tagging constructs [pBA100; KKT19, pBA72; KKT7, pBA68; KKT8, pBA73; KKT9 and pBA75; KKT11 (Akiyoshi and Gull, 2014)] were subcloned into pBA148 using *Xba*I/*Bam*HI. To make pBA1444 (3Flag tagging), PCR was performed using primers BA1995/BA1996 and pEnT6B-Y (Kelly et al., 2007) as a template, then the DNA was digested with *Xba*I and self-ligated. To make N-terminal 3Flag-tagged constructs (pBA1452; KKT4 and pBA1453; KKT7), endogenous gene targeting sequences from pBA71 (KKT4) and pBA72 (KKT7) (Akiyoshi and Gull, 2014) were subcloned into pBA1444 *Xba*I/*Bam*HI sites. To make pBA1613 (N-terminal TY-YFP-tagging construct for KKT7), the coding sequence of KKT7 (4–1932 bp) was amplified with primers BA286/BA2145 from genomic DNA and subcloned into *Xba*I/*Not*I sites of pBA72 (Akiyoshi and Gull, 2014). Site-directed mutagenesis was performed to make pBA2033 using pBA1613 as a starting template and the following primers: BA2156/BA2157 (S23A T27A), BA2184/BA2185 (S34A S36A), BA2186/BA2187 (S65A T67A), BA2188/BA2189 (S148A S150A), BA2620/BA2621 (S257A S263A), BA2622/BA2623 (S317A), BA2624/BA2625 (S490A), BA2616/BA2617 (A27T, to mutate back to wild type), and BA2618/BA2619 (A67T, to mutate back to wild type). To make pBA1806 (N-terminal TY-YFP-tagging construct for KKT2), the coding sequence of KKT2 (4–3780 bp) was amplified with primers BA266/BA2346 from genomic DNA and subcloned into *Hin*dIII/*Not*I sites of pBA67 (Akiyoshi and Gull, 2014), and then site-directed mutagenesis was performed using primers BA2639/BA2640 to make pBA2034. To make pBA346, a DNA fragment for the KKT7 N-terminal domain was amplified using primers BA733/BA734 and cloned into pBA310 *Pac*I/*Afl*II sites. To make pBA347, a DNA fragment for the KKT7 C-terminal domain was amplified using primers BA735/BA736 and cloned into pBA310 *Pac*I/*Afl*II sites. An inducible GFP-NLS-LacI plasmid (pBA795) was made as follows. First, LacI was amplified from pMig75 (Navarro and Gull, 2001) using primers BA1063/BA1064 and cloned into pBA310 using *Asc*I/*Afl*II to make pBA608. Then the DNA fragment containing GFP-NLS-LacI was subcloned into pDex877-GFP-TY using *Nhe*I/*Not*I to make pBA795. A DNA fragment containing the KKT7 N-terminal domain was amplified with primers BA1401/BA1402 and cloned into pBA795 at *Pac*I/*Asc*I site to make pBA891. To make pBA892 (N-terminal TY-tdTomato-tagging vector with hygromycin marker), a DNA fragment of pBA148 that encodes TY-tdTomato was subcloned into pEnT5-Y using *Spe*I/*Xba*I sites. To make N-terminal TY-tdTomato-tagging constructs (pBA919; KKT10 and pBA1103; KKT19), endogenous gene targeting sequences from TY-YFP-tagging constructs [pBA74; KKT10 and pBA100; KKT19 (Akiyoshi and Gull, 2014)] were subcloned into pBA892 using *Xba*I/*Bam*HI.

To make pBA1641 and pBA1513, KKT4^300–488^ or KKT4^463–645^ were amplified from a plasmid that encodes KKT4 using BA2146/BA2149 or BA2037/BA992, and cloned into the pNIC28-Bsa4 expression vector (gift of the Structural Genomics Consortium, Oxford, UK) using a ligation-independent cloning method (Gileadi et al., 2008). To make pBA234 and pBA261, KKT10 or 2Flag-KKT7^2–261^ were amplified from genomic DNA using BA605/BA606 or BA644/BA646, and then cloned into the pNIC28-Bsa4 expression vector. To make pBA607, 6HIS-KKT10 was amplified from a plasmid that encodes KKT10 using BA1061/BA1062 and cloned into pST44 (Tan et al., 2005) using *Xba*I/*Stu*I sites. To make pBA660, KKT7N^2–261^ was amplified from a plasmid that encodes KKT7 using BA1065/BA1068 and cloned into pBA607 using *Eco*RI/*Sac*I sites. To make pBA457, synthetic DNA BAG0′ (GeneArt) that has four translation cassettes for full-length 6HIS-KKT8, KKT9, KKT11, and KKT12 (each gene was codon-optimized for expression in *E. coli*) was subcloned into pST44 using *Xba*I/*Mlu*I sites.

To make pBA1834, site-directed mutagenesis was performed using primers BA2367/BA2368 and pBA1641 as a template. To make pBA1879, site-directed mutagenesis was performed using primers BA2371/BA2372 and pBA1834 as a template. To make pBA1888, site-directed mutagenesis was performed using primers BA2369/BA2370 and pBA1879 as a template. To make pBA2016, KKT4^300–488^ containing S334A S463A S477A was amplified from pBA1888 using primers BA2612/BA2613 and cloned into a vector amplified from pBA1606 using primers BA2614/BA2615. Site-directed mutagenesis was performed using pBA1606 (Llauró et al., 2018) as a template and primers BA2367/BA2368 (S334A), BA2369/BA2370 (S463A), or BA2371/BA2372 (S477A) to make pBA2047, pBA2048, and pBA2049, respectively.

To make pBA805 (KKT1^1–990^-3Flag), pBA806 (KKT1^990–1594^-3Flag), and pBA818 (3Flag-KKT4), KKT1 fragments or KKT4 were amplified using BA1344/BA1345, BA1346/BA1347, or BA1355/BA1356, and cloned into pACEBac2 (Geneva Biotech) using *Xma*I/*Nhe*I sites. These plasmids were integrated into the DH10EmBacY baculoviral genome in DH10EmBacY *E. coli* cells to make pBA822, pBA824, and pBA826 bacmids. Bacmids were purified from *E. coli* using a PureLink HiPure Plasmid Miniprep Kit (Thermo Fisher).

### Cells

All cell lines used in this study were derived from *T. brucei* SmOxP927 procyclic form cells (Poon et al., 2012) and are listed in Table S9. Cells were grown at 28°C in SDM-79 medium (Life Technologies) supplemented with 10% (v/v) heat-inactivated fetal calf serum (Sigma) (Brun and Schönenberger, 1979) with puromycin (Sigma) and appropriate drugs. For induction of RNAi or ectopic expression of GFP-NLS fusion proteins, doxycycline (Sigma) was added to the medium to a final concentration of 1 µg/ml or 10 ng/ml, respectively.

Gene deletions were carried out as described previously (Merritt and Stuart, 2013). To make deletion strains (BAP1004; kkt19Δ, BAP1054; kkt10Δ), a fusion of three PCR products [first, the upstream targeting sequence distal to KKT19 (primers BA1848/BA1849) or KKT10 (primers BA1836/BA1837) amplified from genomic DNA; second, the neomycin marker cassette amplified from pBA183 using primers BA903/BA904; and third, the downstream targeting sequence distal to KKT19 (primers BA1855/BA1856) or KKT10 (primers BA1844/BA1845) amplified from genomic DNA] was transfected into SmOxP927 by electroporation. Transfected cells were selected by addition of 30 µg/ml G418 (Sigma) and cloned by dispensing dilutions into 96-well plates. To make BAP1068 (kkt19Δ/kkt19Δ) and BAP1073 (kkt10Δ/kkt10Δ), a fusion of three PCR products [first, the upstream targeting sequence distal to KKT19 (primers BA1858/BA1859) or KKT10 (primers BA1838/BA1839) amplified from genomic DNA; second, the clonNAT marker cassette amplified from pMig75 using primers BA905/BA906; and third, the downstream targeting sequence distal to KKT19 (primers BA1860/BA1861) or KKT10 (primers BA1842/BA1843) amplified from genomic DNA] was transfected into BAP1004 or BAP1054. Transfected cells were selected by addition of 100 µg/ml nourseothricin/clonNAT (Jena Bioscience) and cloned by dispensing dilutions into 96-well plates. Deletions were checked by PCR. We could not maintain the strain that has 3Flag-KKT4 kkt19Δ/kkt19Δ KKT10 RNAi because cells did not grow well, which is likely due to a small level of leakage of RNAi even in the absence of doxycycline (data not shown). This result suggests that there is a negative genetic interaction between 3Flag–KKT4 and KKT10/19.

For C-terminally YFP-tagged KKT2 (BAP1579), YFP-tagging cassette was amplified from pPOTv7 (Dean et al., 2015) using primers BA2267/BA2268. The PCR product was transfected into SmOxP927 by electroporation. Transfected cells were selected by the addition of 10 µg/ml blasticidin S (Insight biotechnology).

All plasmids were linearized by *Not*I and transfected to trypanosomes by electroporation into an endogenous locus (TY-YFP tagging, TY-tdTomato tagging, 3Flag-6His-YFP tagging, and 3Flag tagging), rDNA locus (pMig96) or 177 bp repeats on minichromosomes (RNAi, GFP-NLS-KKT7N/C, and GFP-NLS-KKT7N-LacI). Transfected cells were selected by the addition of 25 µg/ml hygromycin (Sigma), 10 µg/ml blasticidin S (Insight biotechnology) or 5 µg/ml phleomycin (Sigma).

### Fluorescence microscopy

Cells were fixed with formaldehyde as previously described (Nerusheva and Akiyoshi, 2016). Images were captured at room temperature on a DeltaVision fluorescence microscope (Applied Precision) installed with softWoRx version 5.5 housed in the Oxford Micron facility. Fluorescent images were captured with a CoolSNAP HQ camera using 60× objective lenses (1.42 NA). Typically, 25 optical slices spaced 0.2 µm apart were collected. Maximum intensity projection images were generated by Fiji software (Schindelin et al., 2012).

### Immunoblotting

Cells were harvested by centrifugation (800 ***g***, 5 min) and washed with 1 ml PBS. Then 25% trichloroacetic acid (TCA) was added and mixture was incubated for 5 min on ice, followed by centrifugation at 21,000 ***g*** at room temperature for 1 min. Then cells were washed with ice-cold acetone and the supernatant removed. The pellet was resuspended in 1× LDS sample buffer (Thermo Fisher) with 0.1 M DTT. Denaturation of proteins was performed for 5 min at 95°C.

SDS-PAGE and immunoblots were performed by standard methods using the following mouse monoclonal antibodies: BB2 (anti-TY, 1:100) (Bastin et al., 1996) for TY-YFP-tagged KKT proteins, L8C4 (anti-PFR2, 1:1500) (Kohl et al., 1999) for a loading control and anti-Flag (Sigma, clone M2 F3165, 1:500). Bands were visualized by horseradish-peroxidase-conjugated sheep anti-mouse-IgG antibodies (GE Healthcare) on an ODYSSEY Fc Imaging System (LI-COR).

### Protein purification

For affinity-purification of GFP-NLS-KKT7N and KKT7C from trypanosomes, their expression was induced with 10 ng/ml doxycycline for 24 h. Immunoprecipitation and mass spectrometry were performed essentially as described previously (Nerusheva et al., 2019) at the Advanced Proteomics Facility in University of Oxford using MASCOT (version 2.5.1, Matrix Science). Proteins identified with at least two peptides were considered and shown in Tables S4 and S5.

To identify phosphorylation sites on kinetochore proteins from our previous immunoprecipitation data, the Mascot search engine (version 2.5.1, Matrix Science) was used with the following parameters: up to two missed cleavages were allowed; carbamidomethylation (Cys) was set as a fixed modification; oxidation (Met), phosphorylation (Ser, Thr, Tyr), acetylation (Lys), and N-acetylation (protein) were set as variable modifications; the precursor ion mass tolerance was set to 20 ppm; fragment ion mass tolerance was set to 0.5 Da for LTQ XL-Orbitrap data (Akiyoshi and Gull, 2014; Nerusheva and Akiyoshi, 2016) or 0.02 Da for Q Exactive data (Nerusheva et al., 2019). Phosphopeptides with Mascot score >30 were considered significant and listed in Table S1 (kinetochore proteins only) and Table S2 (all proteins). Table S3 summarizes the phosphorylation sites detected in proteomics studies (Benz and Urbaniak, 2019; Nett et al., 2009; Urbaniak et al., 2013).

Recombinant KKT4 fragments (pBA1413: KKT4^2–114^, pBA1065: KKT4^115–343^, pBA1641: KKT4^300–488^, pBA1513: KKT4^463–645^) were expressed in *E. coli* BL21(DE3) cells and purified as previously described (Llauró et al., 2018) with the following further purification steps. Proteins eluted from TALON beads (Takara Bio) using P500 buffer (50 mM sodium phosphate, pH 7.5, 500 mM NaCl, and 10% glycerol) with 250 mM imidazole were treated with TEV protease tagged with 6HIS tag (Tropea et al., 2009) to remove the 6HIS tag from the KKT4 fragments while being dialyzed into P500 buffer with 5 mM imidazole. Then the protein was applied through TALON beads to get rid of 6HIS-TEV and non-cleaved proteins, and loaded onto a HiPrep Superdex 75 16/60 size exclusion chromatography column (GE Healthcare) pre-equilibrated with 25 mM HEPES pH 7.5, 150 mM NaCl, and 1 mM TCEP. Fractions containing KKT4 fragments were pooled together, concentrated with an Amicon stirred cell using an ultrafiltration disc with 10 kDa cut-off, and stored at −80°C.

Recombinant 6HIS-KKT10 (pBA234), 6HIS-2Flag-KKT7^2–261^ (pBA261), 6HIS-KKT10 KKT7^2–261^ (pBA660), and 6HIS-KKT8, KKT9, KKT11, KKT12 (pBA457) were expressed in Rosetta 2(DE3)pLys *E. coli* cells (Novagen), purified and eluted from TALON beads using a protocol that is previously described (Llauró et al., 2018).

Bacmids (pBA822, pBA824, pBA826) were used to transfect Sf9 cells using Cellfectin II transfection reagent (Thermo Fisher). Sf9 cells were grown in Sf-900 II SFM media (Thermo Fisher). Baculovirus was amplified through three rounds of amplification. 3Flag-tagged proteins were expressed and purified from Sf9 cells using a protocol described previously (Llauró et al., 2018). Protein concentration was determined by protein assay (Bio-Rad).

### *In vitro* kinase assay

Recombinant kinetochore proteins (final concentration: ∼60 µg/ml) mixed with 6HIS-KKT10 (final concentration: 50 µg/ml) in kinase buffer (50 mM Tris-HCl pH 7.4, 1 mM DTT, 25 mM β-glycerophosphate, 5 mM MgCl_2_, 5 µCi [^32^P]ATP, and 10 µM ATP) were incubated at 30°C for 30 min. The reaction was stopped by the addition of LDS sample buffer (Thermo Fisher). The samples were run on an SDS-PAGE gel, which was stained with Coomassie Brilliant Blue R-250 (Bio-Rad) and subsequently dried and used for autoradiography using a Phosphorimager Screen. The signal was detected by an FLA 7000 scanner (GE Healthcare).

## Supplementary Material

Supplementary information

Reviewer comments
