# Supplementary material for: Characterization of unconventional kinetochore kinases KKT10 and KKT19 in Trypanosoma brucei
Source: J Cell Sci. 2020 Apr 29;133(8):jcs240978. doi: 10.1242/jcs.240978 (PMC7197874; doi:10.1242/jcs.240978)
Supplement: Supplementary information [file joces-133-240978-s1.pdf]

Supplemental materials

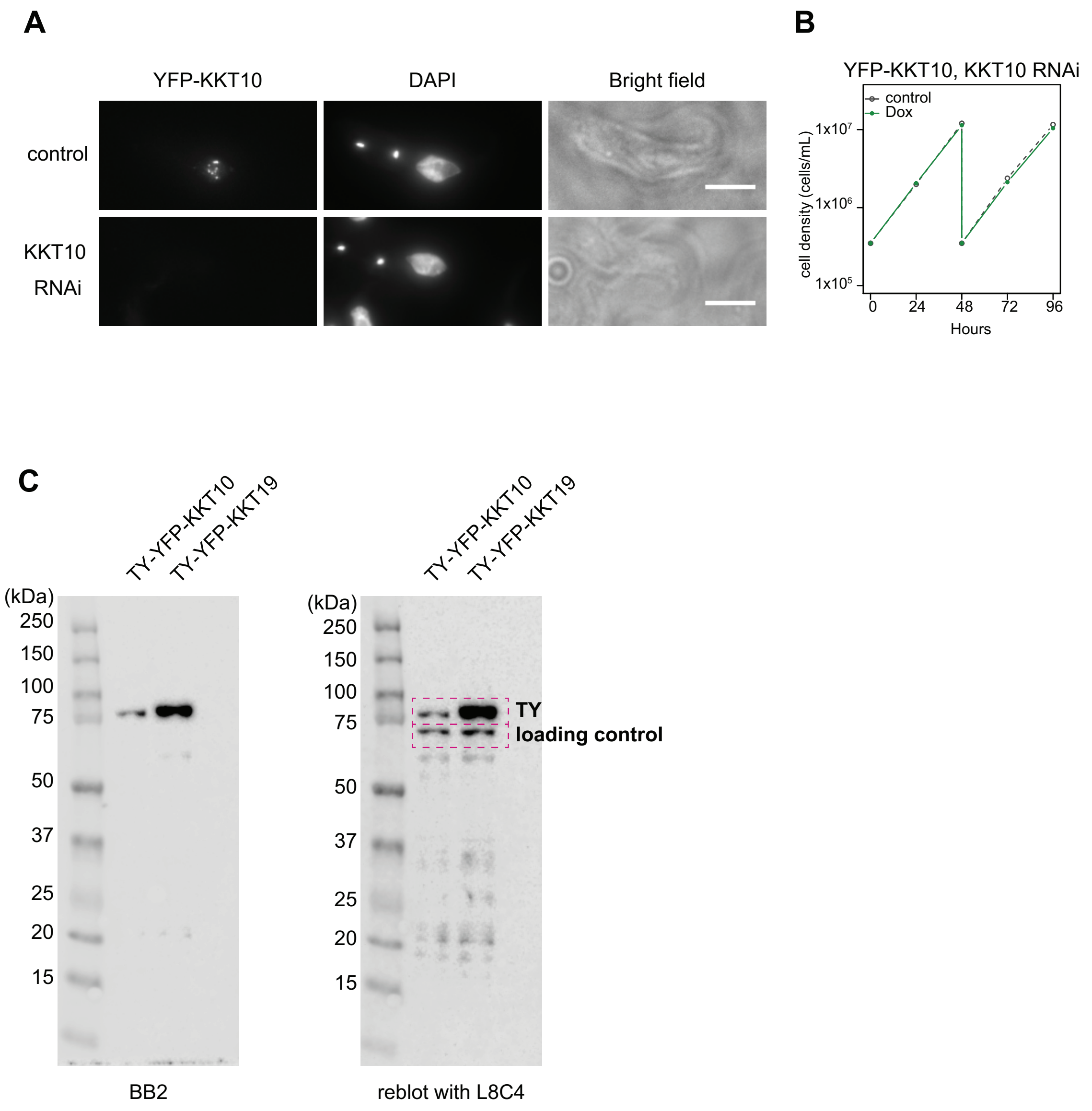

Figure S1. KKT10-specific RNAi does not affect cell growth.

(A) Example of 2K1N cells expressing YFP-KKT10 under KKT10-specific RNAi. Cells were fixed at 24 hours postinduction of RNAi. Control is an uninduced cell culture. Maximum intensity projections are shown. Bars, 5 μm.

(B) Growth curve of YFP-KKT10 with KKT10-specific RNAi. Control is an uninduced cell culture.

(C) Top-bottom gels of cropped immunoblots shown in Fig. 1C.

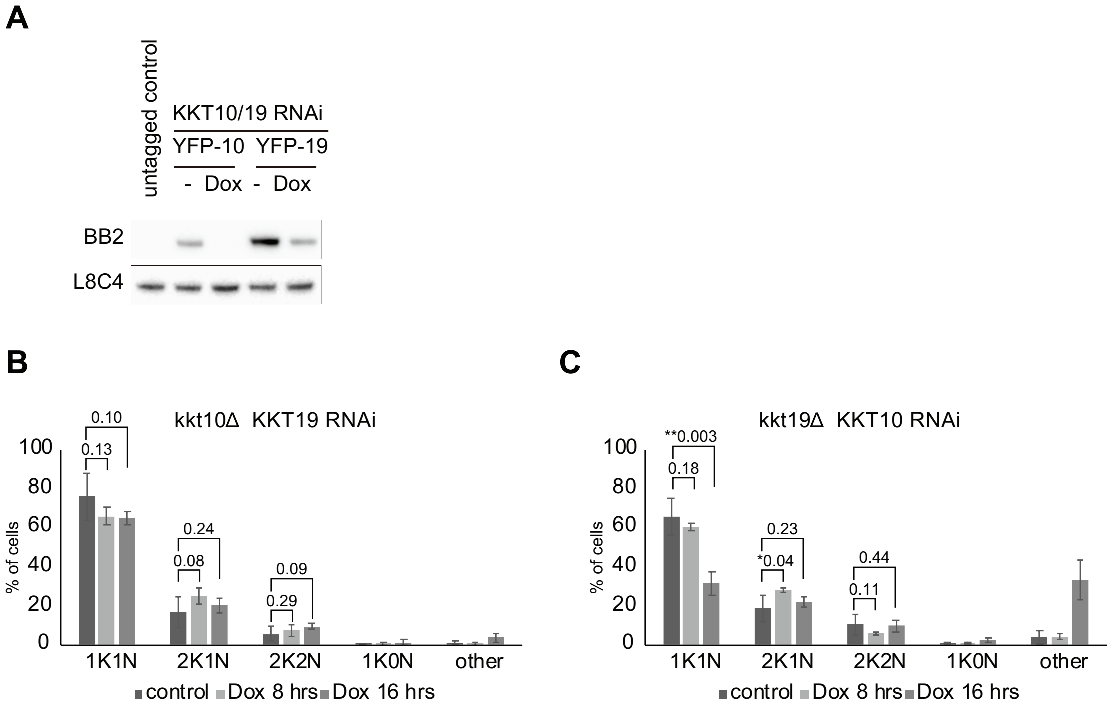

Figure S2. Cell cycle profiles of KKT10/19 depletion.

(A) TY-YFP-tagged KKT10 and KKT19 after KKT10/19 double knockdown RNAi for 24 hours were detected by immunoblotting against the TY tag using BB2 antibodies. PFR2 detected by L8C4 antibodies was used as a loading control. SmOxP9 was used as an untagged control.

(B and C) Quantification of cells with indicated DNA contents. Control is an uninduced cell culture. Error bars represent standard deviation from three independent experiments (n ≥ 314).

Journal of Cell Science • Supplementary information

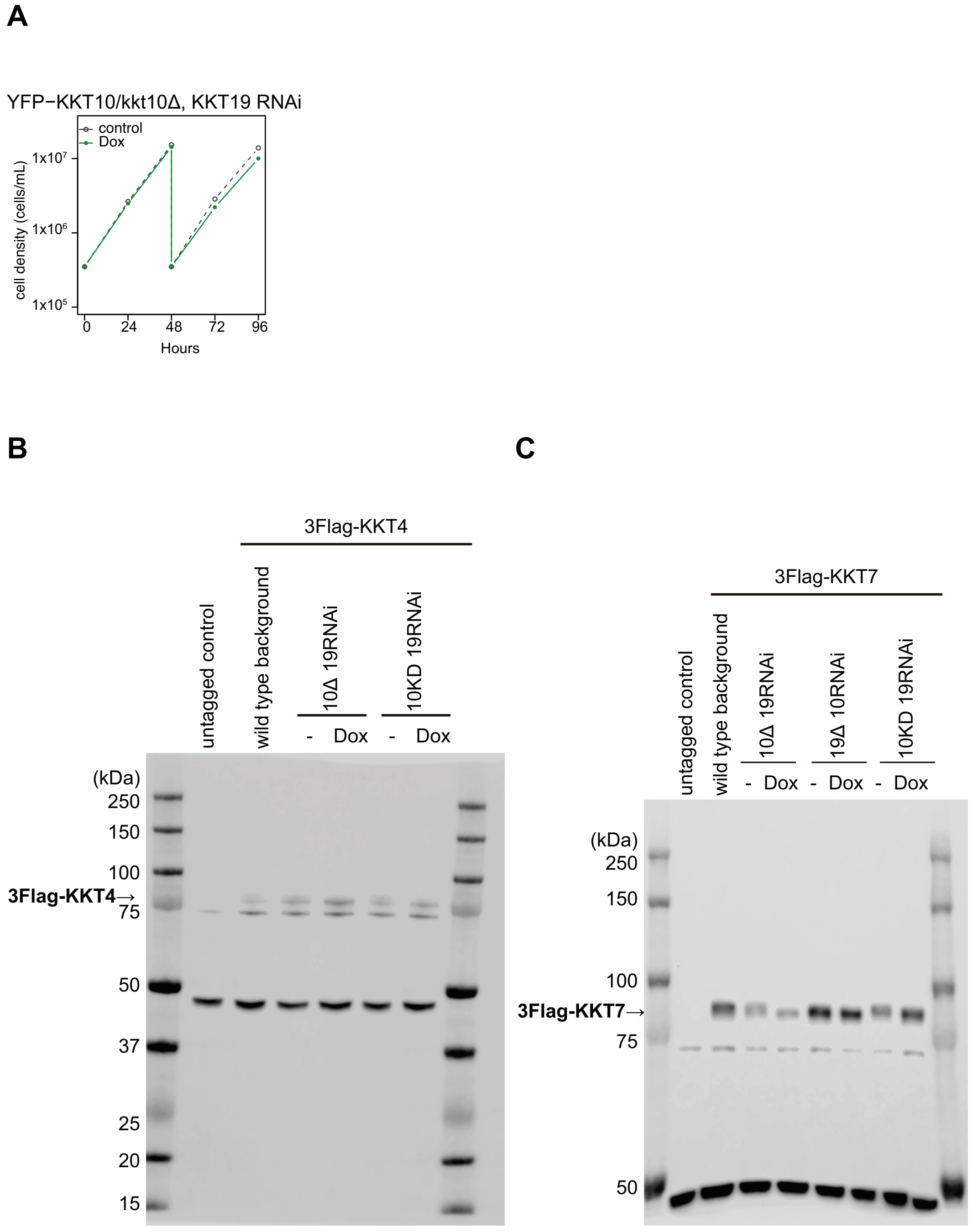

Figure S3. YFP-KKT10^{WT} is fully functional.

(A) Growth curve of YFP-KKT10/kkt10Δ KKT19 RNAi. Control is an uninduced cell culture. Similar results were obtained from three independent experiments.

(B and C) Top-bottom gels of cropped immunoblots shown in Fig. 5B. SmOxP9 was used as an untagged control. 10KD is KKT10^{K158A}.

Journal of Cell Science • Supplementary information

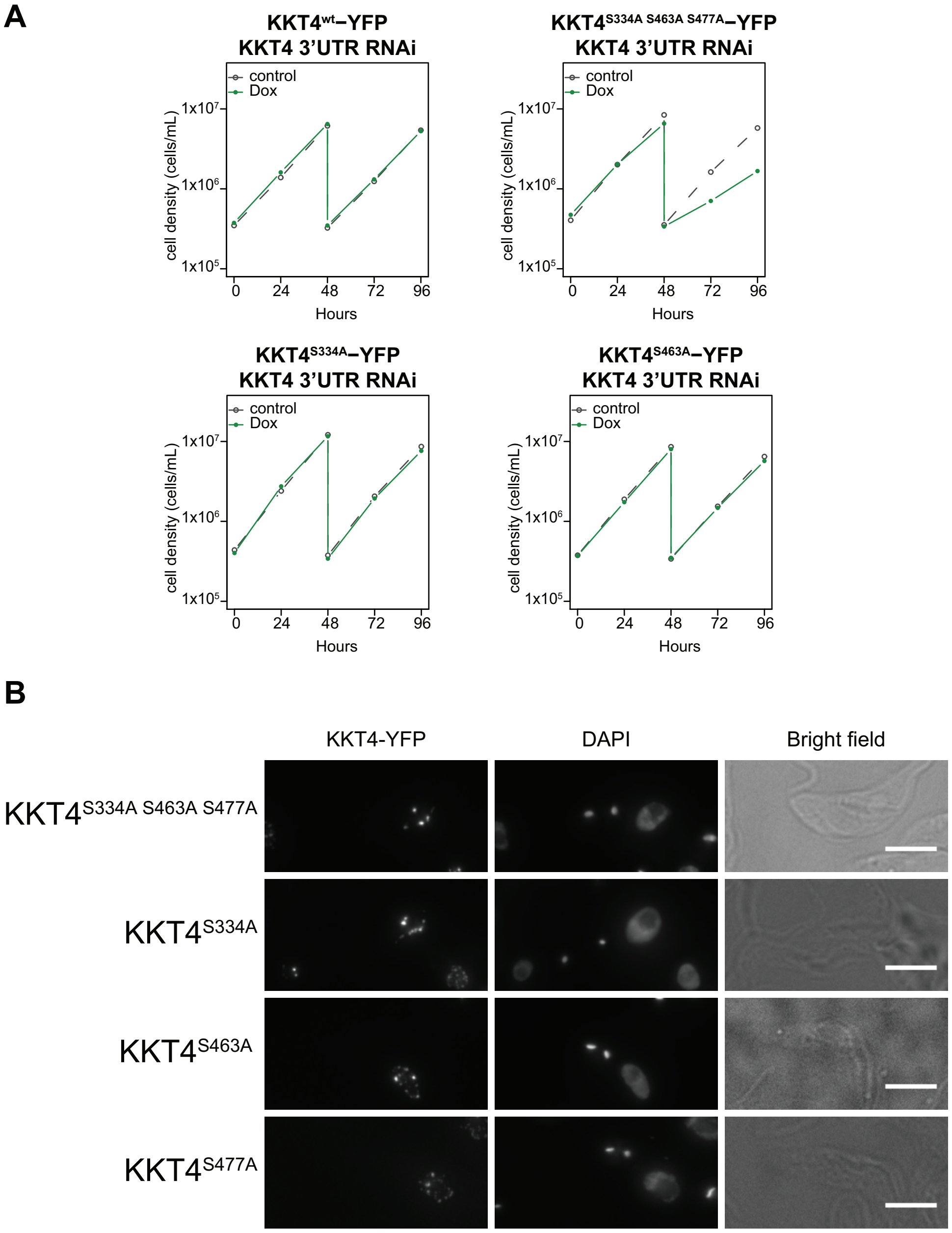

Figure S4. Phospho-deficient KKT4$^{S334A\ S463A\ S477A}$ mutant cannot support cell growth.

(A) Growth curve of KKT4^{wt}-YFP with KKT4 3´UTR RNAi, KKT4$^{S334A\ S463A\ S477A}$-YFP with KKT4 3´UTR RNAi, KKT4^{S334A}-YFP KKT4 with 3´UTR RNAi, and KKT4^{S463A}-YFP with KKT4 3´UTR RNAi. Control is an uninduced cell culture.

(B) KKT4$^{S334A\ S463A\ S477A}$-YFP, KKT4^{S334A}-YFP, KKT4^{S463A}-YFP, and KKT4^{S477A}-YFP localize normally at kinetochores. Bars, 5 μm.

Journal of Cell Science • Supplementary information

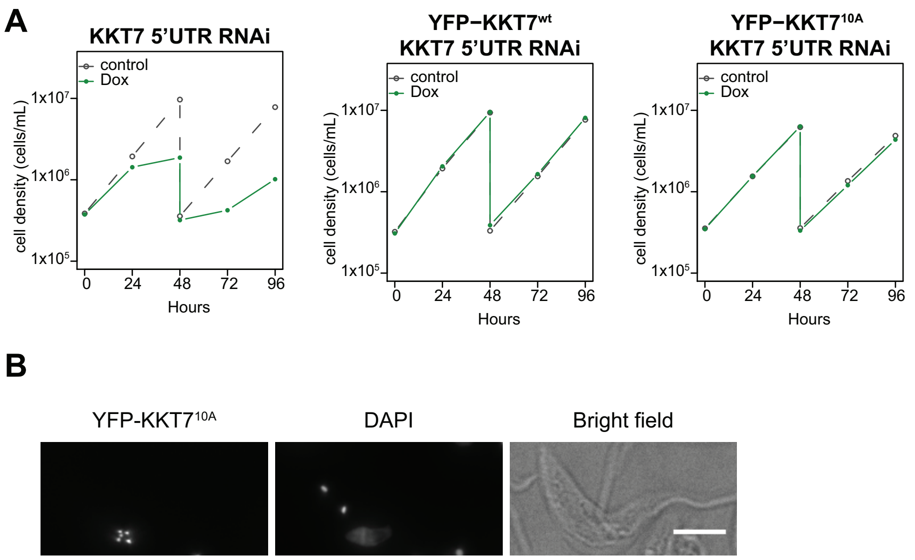

Figure S5. KKT7^{10A} is functional.

(A) Growth curve of KKT7 5´UTR RNAi, YFP-KKT7^{wt} with KKT7 5´UTR RNAi, and YFP-KKT7^{10A} with KKT7 5´UTR RNAi. Control is an uninduced cell culture.

(B) YFP-KKT7^{10A} localizes normally at kinetochores. Bar, 5 μm.

Journal of Cell Science • Supplementary information

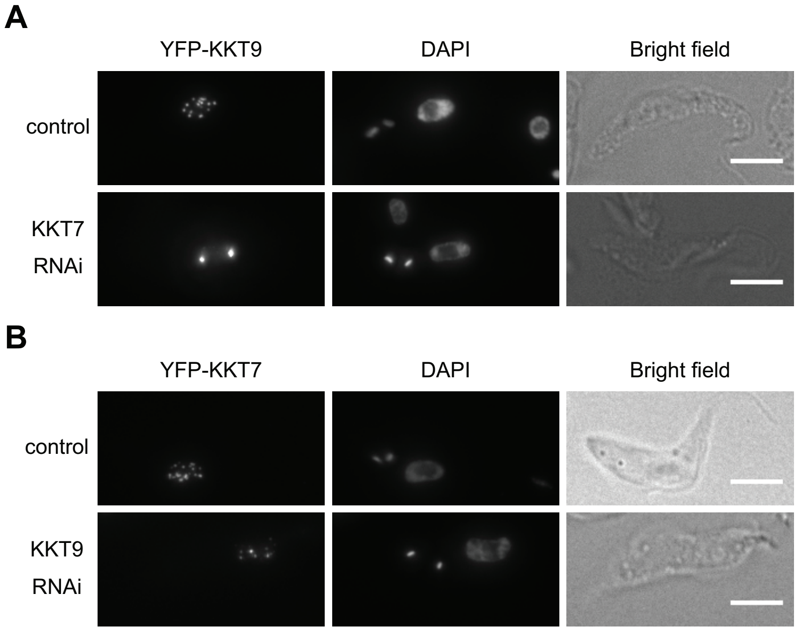

Figure S6. Localization of KKT9 is affected in KKT7-knockdown cells.

(A) YFP-KKT9 in KKT7 RNAi cells. (B) YFP-KKT7 in KKT9 RNAi cells. Cells were fixed at 24 hours postinduction of RNAi and stained with DAPI. Control is an uninduced cell culture. Maximum intensity projections are shown. Bars, 5 μm.

Journal of Cell Science • Supplementary information

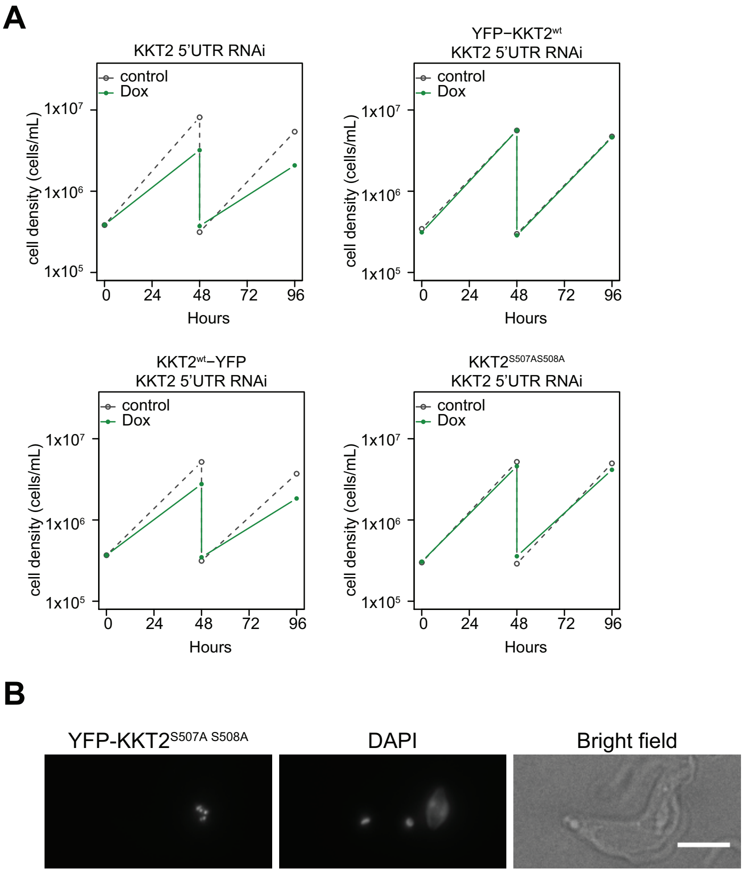

Figure S7. YFP-KKT2$^{S507A\ S508A}$ is functional in procyclic cells.

(A) Growth curve of KKT2 5´UTR RNAi, YFP-KKT2^{wt} with KKT2 5´UTR RNAi, KKT2^{wt}-YFP with KKT2 5´UTR RNAi, and YFP-KKT2^{S507AS508A} with KKT2 5´UTR RNAi. Control is an uninduced cell culture.

(B) YFP-KKT2$^{S507A\ S508A}$ localizes normally at kinetochores. Example of cells expressing YFP-KKT2$^{S507A\ S508A}$.

Journal of Cell Science • Supplementary information

Supplementary dataset

Table S1. Phosphorylation sites on kinetochore proteins identified in our previous immunoprecipitates of kinetochore proteins.

Click here to Download Table S1

Table S2. Raw data containing phosphorylation sites on all proteins identified in our previous immunoprecipitates of kinetochore proteins.

Click here to Download Table S2

Table S3. Phosphorylation sites on kinetochore proteins identified by proteomic studies of *T. brucei* cell extracts.

Click here to Download Table S3

Table S4. List of proteins identified in the immunoprecipitates of YFP-tagged KKT7N by mass spectrometry, Related to Figure 6.

Click here to Download Table S4

Journal of Cell Science • Supplementary information

Table S5. List of proteins identified in the immunoprecipitates of YFP-tagged KKT7C by mass spectrometry, Related to Figure 6.

Click here to Download Table S5

Table S6. List of plasmids and bacmids used in this study.

Click here to Download Table S6

Table S7. List of primers used in this study.

Click here to Download Table S7

Table S8. List of synthetic DNA used in this study.

Click here to Download Table S8

Table S9. List of trypanosome cell lines used in this study.

Click here to Download Table S9

Journal of Cell Science • Supplementary information

Supplementary references

Akiyoshi, B. and Gull, K. (2014). Discovery of unconventional kinetochores in kinetoplastids. *Cell* **156**, 1247–1258.

Dean, S., Sunter, J., Wheeler, R. J., Hodkinson, I., Gluenz, E. and Gull, K. (2015). A toolkit enabling efficient, scalable and reproducible gene tagging in trypanosomatids. *Open Biol* **5**, 140197–140197.

Gileadi, O., Burgess-Brown, N. A., Colebrook, S. M., Berridge, G., Savitsky, P., Smee, C. E. A., Loppnau, P., Johansson, C., Salah, E. and Pantic, N. H. (2008). High throughput production of recombinant human proteins for crystallography. *Methods Mol. Biol.* **426**, 221–246.

Hayashi, H. and Akiyoshi, B. (2018). Degradation of cyclin B is critical for nuclear division in *Trypanosoma brucei*. *Biol Open* **7**, bio031609.

Kelly, S., Reed, J., Kramer, S., Ellis, L., Webb, H., Sunter, J., Salje, J., Marinsek, N., Gull, K., Wickstead, B., et al. (2007). Functional genomics in *Trypanosoma brucei*: a collection of vectors for the expression of tagged proteins from endogenous and ectopic gene loci. *Molecular and Biochemical Parasitology* **154**, 103–109.

Llauró, A., Hayashi, H., Bailey, M. E., Wilson, A., Ludzia, P., Asbury, C. L. and Akiyoshi, B. (2018). The kinetoplastid kinetochore protein KKT4 is an unconventional microtubule tip–coupling protein. *J. Cell Biol.* **217**, 3886–3900.

Navarro, M. and Gull, K. (2001). A pol I transcriptional body associated with VSG mono-allelic expression in *Trypanosoma brucei*. *Nature* **414**, 759–763.

Landeira, D. and Navarro, M. (2007). Nuclear repositioning of the VSG promoter during developmental silencing in *Trypanosoma brucei*. *J. Cell Biol.* **176**, 133–139.

Nerusheva, O. O. and Akiyoshi, B. (2016). Divergent polo box domains underpin the unique kinetoplastid kinetochore. *Open Biol* **6**, 150206.

Poon, S. K., Peacock, L., Gibson, W., Gull, K. and Kelly, S. (2012). A modular and optimized single marker system for generating *Trypanosoma brucei* cell lines expressing T7 RNA polymerase and the tetracycline repressor. *Open Biol* **2**, 110037–110037.

Tan, S., Kern, R. C. and Selleck, W. (2005). The pST44 polycistronic expression system for producing protein complexes in *Escherichia coli*. *Protein Expr. Purif.* **40**, 385–395.

Wickstead, B., Ersfeld, K. and Gull, K. (2002). Targeting of a tetracycline-inducible expression system to the transcriptionally silent minichromosomes of *Trypanosoma brucei*. *Molecular and Biochemical Parasitology* **125**, 211–216.

Journal of Cell Science • Supplementary information
