# Supplementary material for: Characterization of unconventional kinetochore kinases KKT10 and KKT19 in Trypanosoma brucei
Source: J Cell Sci. 2020 Apr 29;133(8):jcs240978. doi: 10.1242/jcs.240978 (PMC7197874; doi:10.1242/jcs.240978)
Supplement: Reviewer comments [file joces-133-240978_review_history.pdf]

Journal of Cell Science

Characterization of unconventional kinetochore kinases KKT10 and KKT19 in *Trypanosoma brucei*

Midori Ishii and Bungo Akiyoshi
DOI: 10.1242/jcs.240978

Editor: David Glover

Review timeline
Original submission: 25 October 2019
Editorial decision: 25 November 2019
First revision received: 31 January 2020
Accepted: 2 March 2020

Original submission

First decision letter

MS ID#: JOCES/2019/240978

MS TITLE: Characterization of unconventional kinetochore kinases KKT10/19 in *Trypanosoma brucei*

AUTHORS: Midori Ishii and Bungo Akiyoshi
ARTICLE TYPE: Research Article

We have now reached a decision on the above manuscript.

To see the reviewers' reports and a copy of this decision letter, please go to: https://submit-jcs.biologists.org and click on the 'Manuscripts with Decisions' queue in the Author Area. (Corresponding author only has access to reviews.)

As you will see, the reviewers raise a number of substantial criticisms that prevent me from accepting the paper at this stage. They suggest, however, that a revised version might prove acceptable, if you can address their concerns. If you think that you can deal satisfactorily with the criticisms on revision, I would be pleased to see a revised manuscript. We would then return it to the reviewers.

Please ensure that you clearly highlight all changes made in the revised manuscript. Please avoid using 'Tracked changes' in Word files as these are lost in PDF conversion.

I should be grateful if you would also provide a point-by-point response detailing how you have dealt with the points raised by the reviewers in the 'Response to Reviewers' box. Please attend to all of the reviewers' comments. If you do not agree with any of their criticisms or suggestions please explain clearly why this is so.

Reviewer 1

Advance summary and potential significance to field

Although many established eukaryotic species used for experimental studies contain largely similar kinetochore structures, Trypanosomes represent an important outlier with no recognizable similarities in terms of kinetochore components. Recent work from the Aikyoshi lab has made

© 2020. Published by The Company of Biologists under the terms of the Creative Commons Attribution License (https://creativecommons.org/licenses/by/4.0/).

substantial progress in identifying Trypanosome kinetochore components, and beginning to define their association, organization and functional contributions to chromosome segregation. This paper represents a great additional step towards defining the molecular mechanisms that underlie kinetochore function in this organism with a particular focus on the kinase subunits KKT10 and KK19. This paper includes an elegant combination of genetics (to knockout these kinases), functional perturbations, cell biological analysis of localization and phenotypes, structure-function analysis (to define key domains and test the role of kinase activity), and biochemistry to reconstitute key complexes and interactions.

This is a strong paper that is a great fit for the Journal of Cell Science, and I support its publication. Overall the experiments are clear, well conducted, and appear carefully controlled, providing a diversity of information and advances.

Comments for the author

Given the focus of this paper on key, novel kinases that are kinetochore localized, it would be helpful to have a bit more information regarding potential substrates. The work in this paper provides Western blots to suggest that KKT4 and KKT7 undergo a phosphorylation-dependent shift in SDS-PAGE gels that requires the KKT10/19 kinases. However, given the extensive mass spectrometry that the Aikyoshi lab has conducted to identify Trypanosome proteins, I was a little surprised that they didn't make an attempt to map phosphorylation sites in vivo (some of this should already be present in their mass spec data). Similarly, given their ability to purify these kinases and some of their interaction partners recombinantly, it would be nice to try an in vitro kinase assay. I absolutely realize that some of these experiments may be a long term effort that will extend to future papers, including the ability to mutate sites within KKT10/19 substrates to test their consequences. However, there would be a lot of value for any additional experiments that would evaluate the kinase activity and substrates for these exciting and novel kinetochore kinases.

Reviewer 2

Advance summary and potential significance to field

In their previous studies, the authors' group identified a number of unconventional kinetochore components in T. brucei. In this manuscript, they characterized unconventional kinetochore components, KKT10 and KKT19 kinases. They found that KKT10/19 localize at kinetochores in metaphase and disappear at the anaphase onset. KKT10/19 has redundant function in efficient metaphase-to-anaphase transition, which is dependent on their kinase activity. Moreover, they suggest that phosphorylation of other kinetochore components KKT4/7 is dependent on KKT10/19. Furthermore, KKT10 recruitment to the kinetochore is dependent on KKT7 and the KKT8 complex. Overall, their results support well the conclusions in the paper except for a few minor points (see below). I recommend publication of this manuscript in JCS once the issues below are addressed.

Comments for the author

Fig 1F. They should explain more clearly how nuclear morphology is abnormal in anaphase with the double mutants. For example, in the DAPI image at bottom, two nuclei seem to be separated well; so, how they evaluate that the nuclear morphology is abnormal there?

Fig 2D. The mobility shifts of KKT4 and KKT7 are indeed consistent with their KKT10/19-dependent phosphorylation. However, mobility shifts may also occur due to other protein modifications. In addition, the result does not provide evidence that KKT4/7 are direct substrates of KKT10/19. If technically feasible, the authors should try in vitro phosphorylation of KKT4/7 (or their fragments) by KKT10/19. However, if it is difficult to obtain recombinant proteins for in vitro kinase assays, it is understandable that authors cannot add such data.

Fig 5G and Fig 6B (bottom). There are still bright dots of YFP-KKT10 in the nuclei. Do they reflect residual kinetochore localization of KKT10? At present, the only result in these figures is one representative image.

© 2020. Published by The Company of Biologists under the terms of the Creative Commons Attribution License (https://creativecommons.org/licenses/by/4.0/).

Some quantitative data will be a good addition to the figures (e.g. cell counting as in Fig 1G).

Reviewer 3

Advance summary and potential significance to field

In this work, two kinetochore-associated kinases KKT10 and KKT19 (also known as CLK1 and CLK2 in other studies) are characterized for their functions in the procyclic form of Trypanosoma brucei. The functions of KKT10 and KKT19 in the procyclic cells appear to differ from those previously reported in the blood stream form cells. There are some interesting observations, but without further mechanistic insight to these life cycle-specific differences, these finding are rather incremental. The dependence of KKT10 kinetochore localization on KKT7 and KKT8 complex is interesting. While the evidence for KKT10 kinase activity on KKT7 is rather weak, the interaction between KKT10/19 and KKT7 is well established in this study. This interaction, while solid, would be more interesting and significant if complemented with high-resolution information on their spatial and temporal organization during mitosis.

Comments for the author

From Fig. 5, it seems that KKT7N was sufficient to recruit KKT10/19 ectopically possibly through their direct interaction. However in Fig. 6, KKT10 localization was shown to be also dependent on the KKT8 complex. Why is this? Is KKT8 complex required for KKT7 localization? During mitosis, do KKT8 complex, KKT7 and KKT10/19 localize to the kinetochores following a specific order? Does KKT7 interact with KKT8 complex too?

It shall be specified in the abstract or the title, that this work focused on procylic form of T. brucei.

Fig. 4 is a systematic investigation of the kinetochore localization of other KKT components in cells lacking KKT10/19. While the images are nicely presented, this figure lacks quantitative information. Could there be changes in number and/or intensity of kinetochore foci upon KKT10/19 depletion?

First revision

Author response to reviewers' comments

Responses to reviewers' comments

First of all, we thank all the reviewers for providing constructive comments on our manuscript. Our point-by-point responses to their comments are as follows (shown in blue).

Reviewer 1 Comments for the Author:
Given the focus of this paper on key, novel kinases that are kinetochore localized, it would be helpful to have a bit more information regarding potential substrates. The work in this paper provides Western blots to suggest that KKT4 and KKT7 undergo a phosphorylation-dependent shift in SDS-PAGE gels that requires the KKT10/19 kinases. However, given the extensive mass spectrometry that the Akiyoshi lab has conducted to identify Trypanosome proteins, I was a little surprised that they didn't make an attempt to map phosphorylation sites in vivo (some of this should already be present in their mass spec data).

As suggested, we analyzed phosphorylation sites detected by mass spectrometry of our previous immunoprecipitates of kinetochore proteins (Table S1, S2). We also summarized all the phosphorylation sites on kinetochore proteins detected in published proteomic studies of *T. brucei* cell extracts (Table S3). For kinetochore proteins, we also analyzed whether the phosphorylation

© 2020. Published by The Company of Biologists under the terms of the Creative Commons Attribution License (https://creativecommons.org/licenses/by/4.0/).

sites match the KKT10/19 consensus phosphorylation motif (RxxS) to try to determine which sites might be phosphorylated by KKT10/19.

Similarly, given their ability to purify these kinases and some of their interaction partners recombinantly, it would be nice to try an in vitro kinase assay. I absolutely realize that some of these experiments may be a long term effort that will extend to future papers, including the ability to mutate sites within KKT10/19 substrates to test their consequences. However, there would be a lot of value for any additional experiments that would evaluate the kinase activity and substrates for these exciting and novel kinetochore kinases.

As suggested, we performed in vitro kinase assays using several recombinant proteins and found that KKT4 and KKT7 were strongly phosphorylated by KKT10 in vitro. We added this result in Fig. 5A and 5C. We then tested KKT4 fragments and found that KKT4$^{300-488}$ was the most strongly phosphorylated. By mutating three serines in this region that match the KKT10 phosphorylation motif, we found that the KKT4^{S477A} mutant causes a growth defect when wild-type KKT4 was depleted (Fig. 5D). We also mutated all serines in KKT7 that match the KKT10 phosphorylation motif but the KKT7^{10A} mutant did not show any obvious growth defect (Fig. S5).

Reviewer 2 Comments for the Author:
Fig 1F. They should explain more clearly how nuclear morphology is abnormal in anaphase with the double mutants. For example, in the DAPI image at bottom, two nuclei seem to be separated well; so, how they evaluate that the nuclear morphology is abnormal there?

In wild-type anaphase cells, daughter nuclei appear as a smooth shape and there is no significant lagging DNA in between them. In contrast, lagging chromosomes and/or elongated nuclear signals were often observed upon depletion of KKT10/19. We added this explanation in the figure legend. Furthermore, we added other images into Fig. 1F to show more examples.

Fig 2D. The mobility shifts of KKT4 and KKT7 are indeed consistent with their KKT10/19-dependent phosphorylation. However, mobility shifts may also occur due to other protein modifications. In addition, the result does not provide evidence that KKT4/7 are direct substrates of KKT10/19. If technically feasible, the authors should try in vitro phosphorylation of KKT4/7 (or their fragments) by KKT10/19. However, if it is difficult to obtain recombinant proteins for in vitro kinase assays, it is understandable that authors cannot add such data.

As we mentioned above, we added in vitro kinase assay data (Fig. 5A and 5C), showing that KKT4 and KKT7 can be phosphorylated by KKT10 in vitro.

Fig 5G and Fig 6B (bottom). There are still bright dots of YFP-KKT10 in the nuclei. Do they reflect residual kinetochore localization of KKT10? At present, the only result in these figures is one representative image. Some quantitative data will be a good addition to the figures (e.g. cell counting as in Fig 1G).

In control 2K1N cells, YFP-KKT10 has multiple dots with little diffuse nuclear signal. In contrast, the number of dots dramatically decreases (typically up to two) and diffuse nuclear signal increases in KKT7, KKT9, or KKT11 RNAi cells. It is true that these bright dots might reflect residual kinetochore localization of KKT10, but these results strongly suggest that the kinetochore localization of KKT10 is affected in these cells. We added this point in line 235-240. As for quantitative data, we mentioned in figure legends that “Similar results were obtained in 88% of 2K1N cells (n = 26).” for Fig. 6G (previously Fig. 5G), and “Similar results were obtained in more than 75% of 2K1N cells (n ≥ 16).” for Fig. 7B (previously Fig. 6B).

Reviewer 3:
While the evidence for KKT10 kinase activity on KKT7 is rather weak,

As we mentioned above, we have performed in vitro kinase assays and shown that KKT4 and KKT7 are phosphorylated by KKT10 in vitro. Although we failed to find any obvious defect in a KKT7 phospho-deficient mutant, we found that the KKT4^{S477A} mutant fails to support normal cell growth.

© 2020. Published by The Company of Biologists under the terms of the Creative Commons Attribution License (https://creativecommons.org/licenses/by/4.0/).

The interaction between KKT10/19 and KKT7 is well established in this study. This interaction, while solid, would be more interesting and significant if complemented with high-resolution information on their spatial and temporal organization during mitosis.

We feel that it is beyond the scope of our manuscript to determine the spatial and temporal organization of KKT7-KKT10/19 by high-resolution microscopy (please see below).

Reviewer 3 Comments for the Author:
From Fig. 5, it seems that KKT7N was sufficient to recruit KKT10/19 ectopically, possibly through their direct interaction. However in Fig. 6, KKT10 localization was shown to be also dependent on the KKT8 complex. Why is this? Is KKT8 complex required for KKT7 localization? During mitosis, do KKT8 complex, KKT7 and KKT10/19 localize to the kinetochores following a specific order? Does KKT7 interact with KKT8 complex too?

We examined YFP-KKT7 in KKT9 RNAi cells and found that kinetochore localization of KKT7 was not affected by KKT9 RNAi (Fig. S6). We also examined YFP-KKT9 in KKT7 RNAi cells and found that kinetochore localization of KKT9 depends on KKT7 (Fig. S6). These results show that although KKT7 directly interacts with KKT10 in vitro, the KKT8 complex is essential for the kinetochore localization of KKT10 in vivo. We attempted to test the interaction between the KKT8 complex and KKT10 as well as that between the KKT8 complex and KKT7 using a polycistronic expression system in bacterial cells but failed to obtain positive results thus far (possibly due to protein insolubility problems). Further studies will be needed to understand how the KKT8 complex promotes the kinetochore localization of KKT10.
Our previous analysis using fixed cells showed that KKT7 localizes at kinetochores from S phase until the end of anaphase, while KKT10 and KKT8 complex components localize from S phase and disappear at the onset of anaphase. We are currently trying to establish multi-color live-cell imaging of kinetochore proteins in *T. brucei* to image the recruitment of each kinetochore protein in higher time resolution, but it is still a challenge for us and we therefore think it is beyond the scope of our manuscript.

It shall be specified in the abstract or the title, that this work focused on procylic form of T. brucei.

As suggested, we specified it in the abstract.

Fig. 4 is a systematic investigation of the kinetochore localization of other KKT components in cells lacking KKT10/19. While the images are nicely presented, this figure lacks quantitative information. Could there be changes in number and/or intensity of kinetochore foci upon KKT10/19 depletion?

We obtained similar results in more than 35 cells in each cell line. We included this information into the figure legend (Fig. 3 in the revised manuscript). As cell cycle proceeds, the intensity of kinetochore dots in wild-type cells dramatically changes (partly due to an apparent clustering of kinetochores caused by an unknown mechanism). The fact that we do not know the precise cell cycle timing of individual cells, especially in KKT10/19 RNAi cells, makes it impractical for us to perform meaningful quantification of the intensity of kinetochore dots. Counting the number of dots would require us to perform super-resolution microscopy on each experimental condition, which is beyond the scope of our manuscript.

<u>Second decision letter</u>

MS ID#: JOCES/2019/240978

MS TITLE: Characterization of unconventional kinetochore kinases KKT10/19 in *Trypanosoma brucei*

AUTHORS: Midori Ishii and Bungo Akiyoshi
ARTICLE TYPE: Research Article

© 2020. Published by The Company of Biologists under the terms of the Creative Commons Attribution License (https://creativecommons.org/licenses/by/4.0/).

I am happy to tell you that your manuscript has been accepted for publication in Journal of Cell Science, pending standard ethics checks.

Reviewer 1

Advance summary and potential significance to field

For this revised version, the authors have conducted additional experiments to evaluate the phosphorylation state of the KKT proteins using mass spectrometry, have tested direct in vitro phosphorylation using kinase assays, and have tested phosphorylation site mutants in KKT proteins. Each of these does a great job of responding to the previous reviewer comments and are valuable additions to the paper. Based on these changes, I fully support its publication in the Journal of Cell Science.

Comments for the author

Nice job!

Reviewer 2

Advance summary and potential significance to field

In their previous studies, the authors' group identified a number of unconventional kinetochore components in T. brucei. In this manuscript, they characterized unconventional kinetochore components, KKT10 and KKT19 kinases. They found that KKT10/19 localizes at kinetochores in metaphase and disappears at the anaphase onset. KKT10/19 has redundant function in efficient metaphase-to-anaphase transition, which is dependent on their kinase activity. Moreover, phosphorylation of other kinetochore components KKT4/7 is dependent on KKT10/19. Furthermore, KKT10 recruitment to the kinetochore is dependent on KKT7 and the KKT8 complex. Overall, the paper has successfully characterized unconventional kinetochore kinases in T. bruce, providing useful information to researchers in the field.

Comments for the author

The authors have addressed all my points in the revised manuscript. I support publication of this paper in JCS.

Reviewer 3

Advance summary and potential significance to field

The revision was done thoroughly.

Comments for the author

I have no further comments.

© 2020. Published by The Company of Biologists under the terms of the Creative Commons Attribution License (https://creativecommons.org/licenses/by/4.0/).
